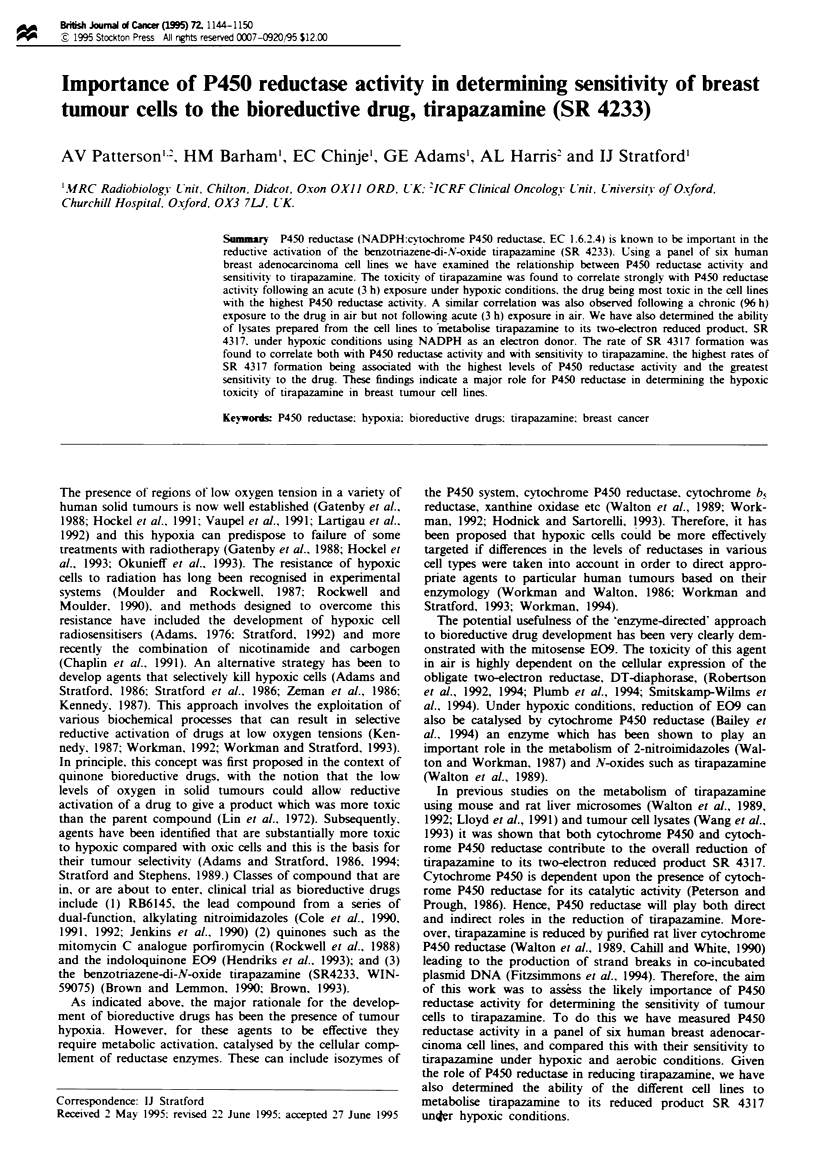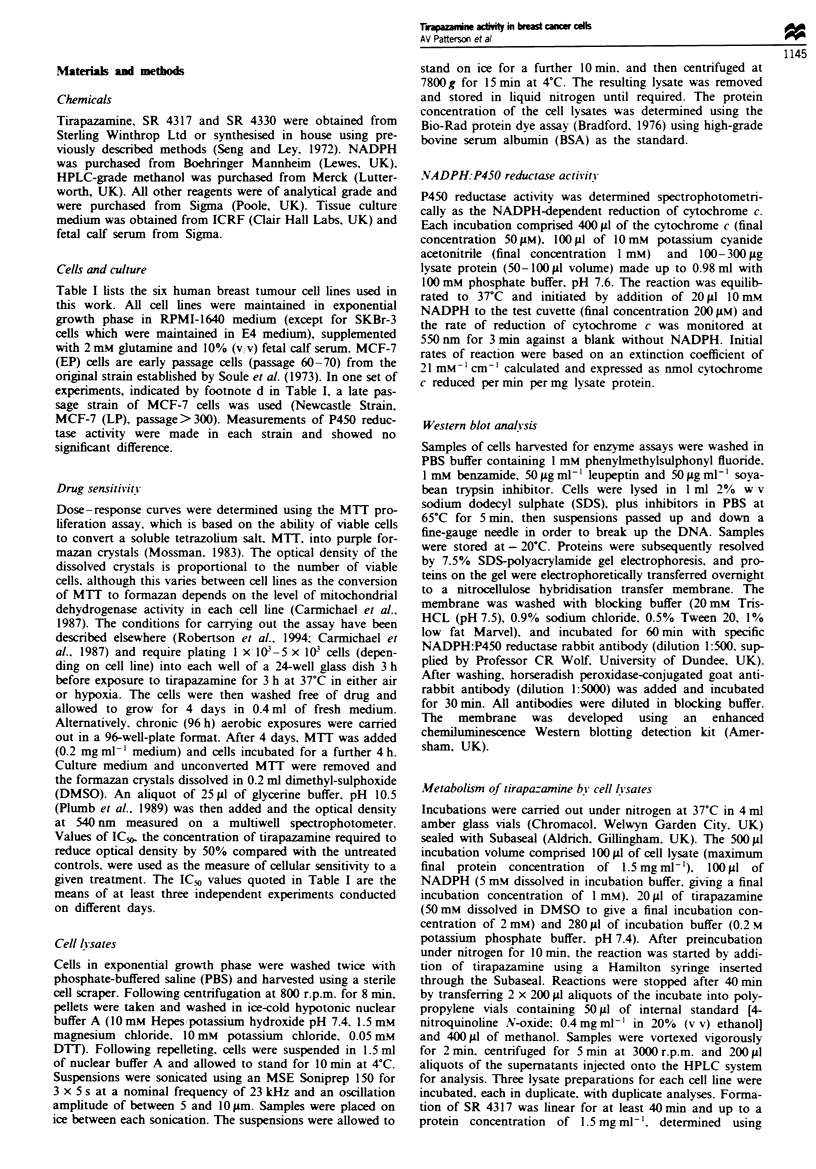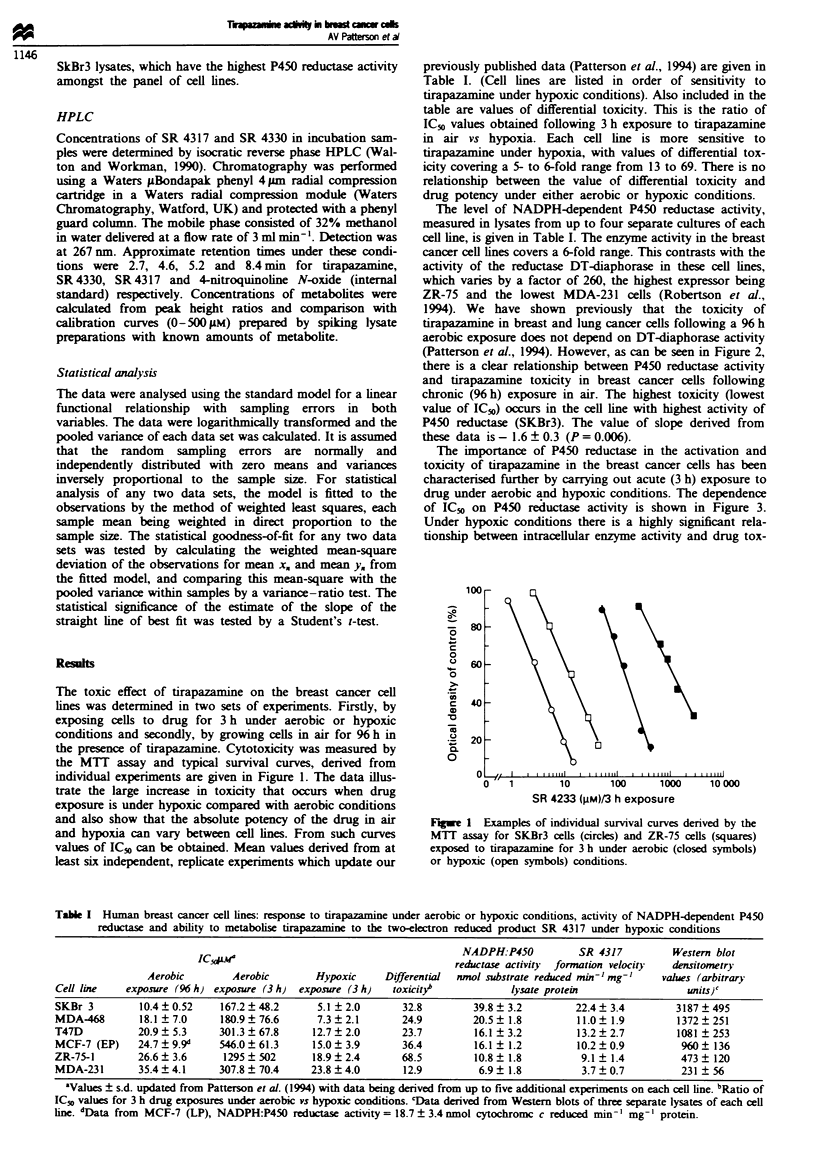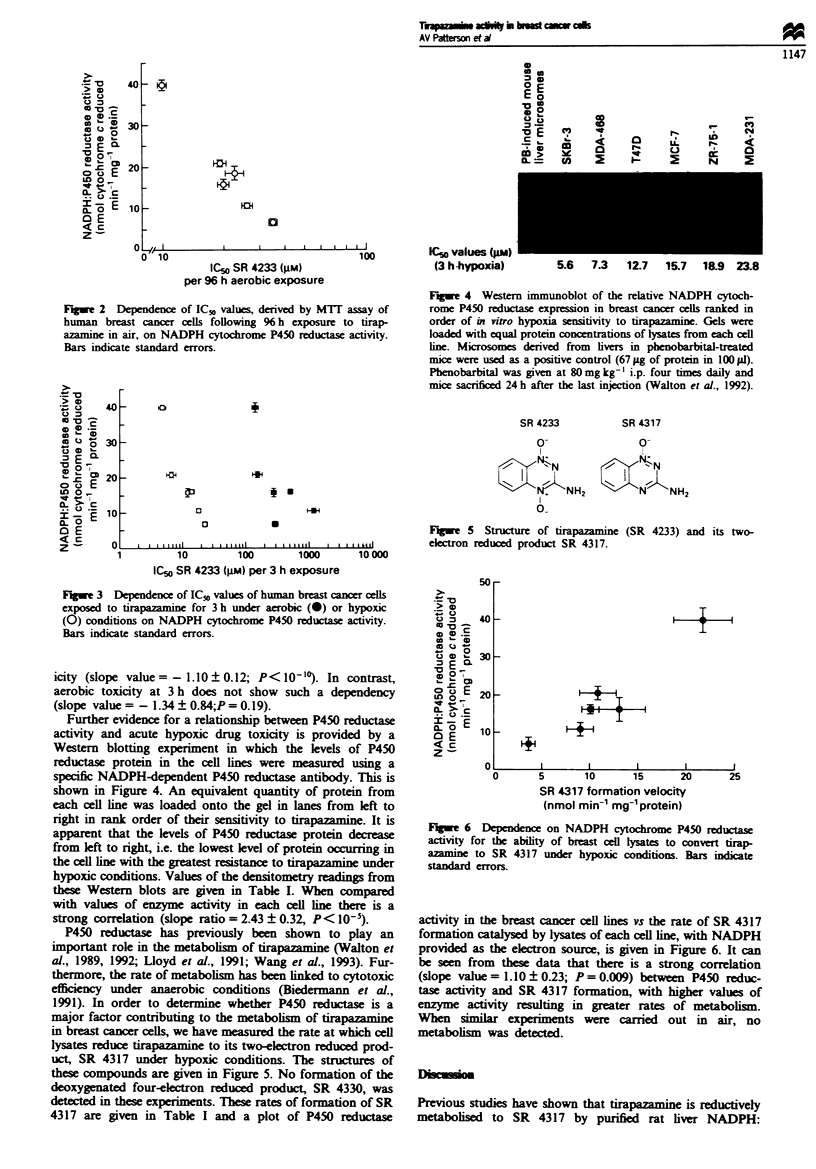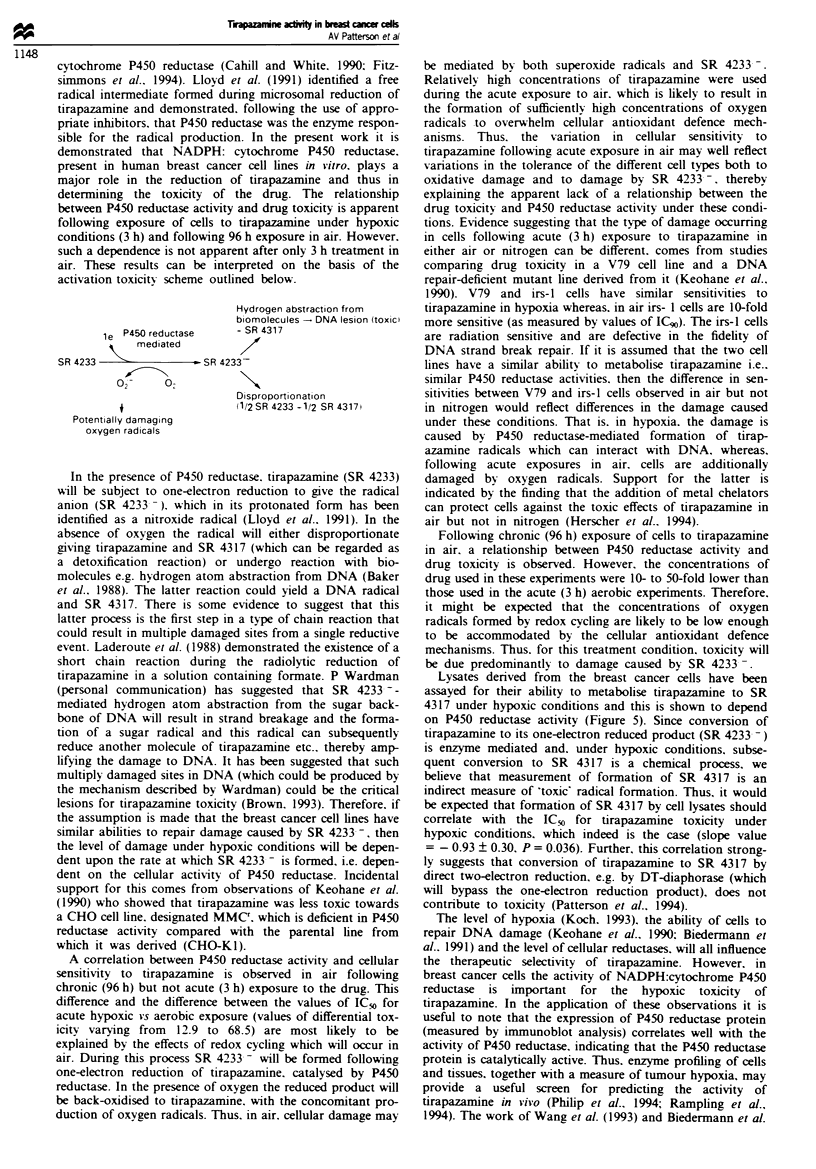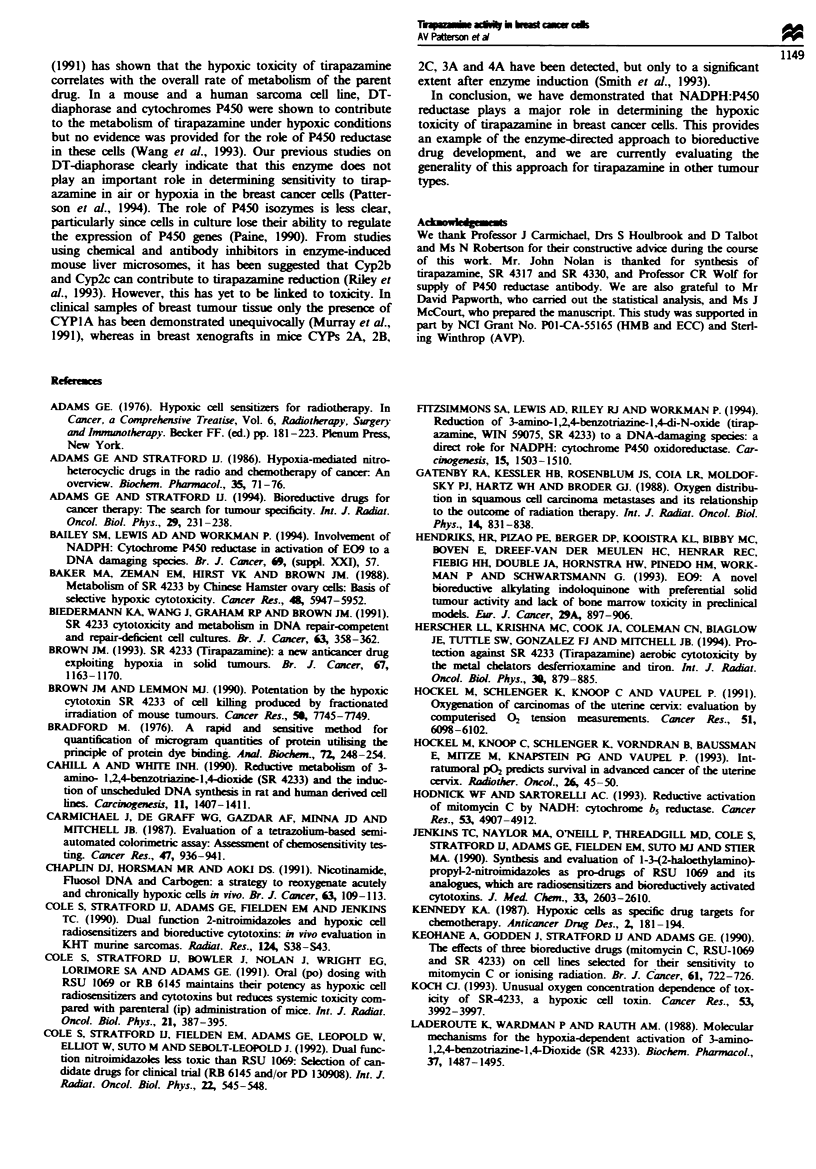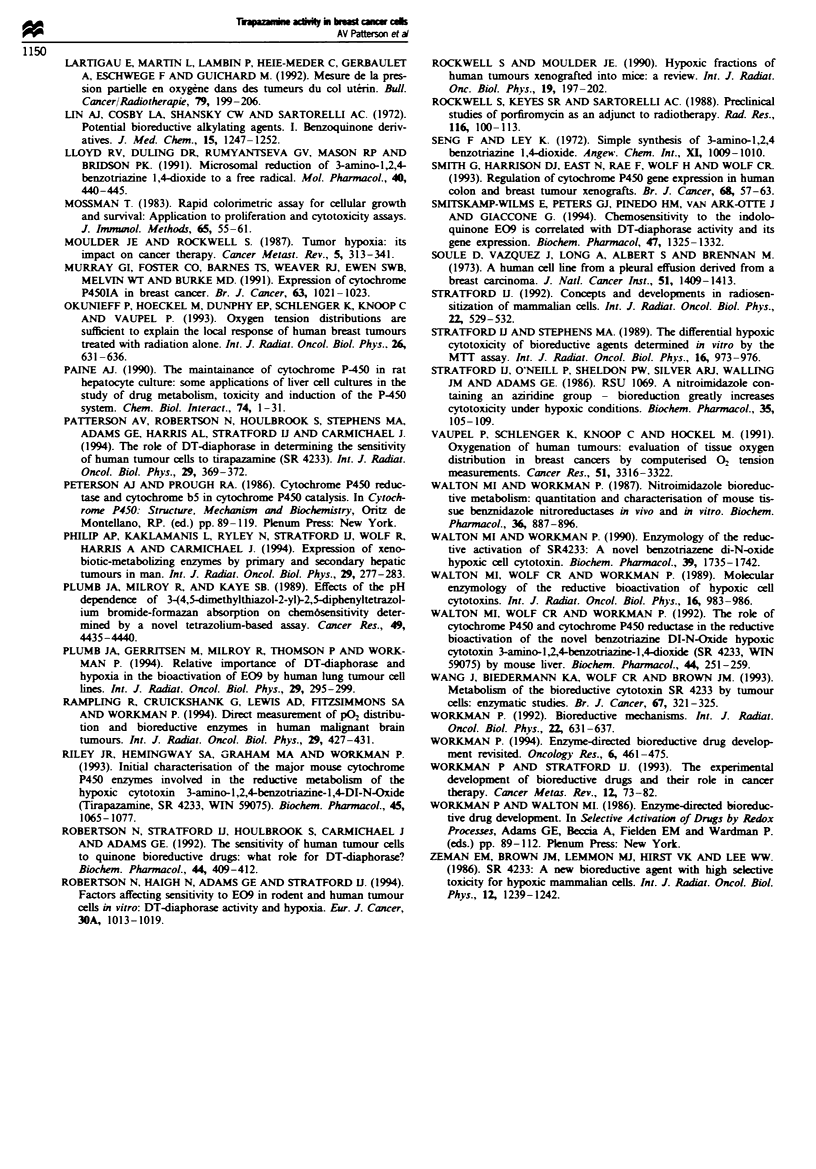# Importance of P450 reductase activity in determining sensitivity of breast tumour cells to the bioreductive drug, tirapazamine (SR 4233).

**DOI:** 10.1038/bjc.1995.478

**Published:** 1995-11

**Authors:** A. V. Patterson, H. M. Barham, E. C. Chinje, G. E. Adams, A. L. Harris, I. J. Stratford

**Affiliations:** MRC Radiobiology Unit, Chilton, Didcot, Oxon, UK.

## Abstract

**Images:**


British Journal of Cancer (1995) 72, 1144–1150

© 1995 Stockton Press All rights reserved 0007-0920/95 $12.00

### Importance of P450 reductase activity in determining sensitivity of breast tumour cells to the bioreductive drug, tirapazamine (SR 4233)

AV Patterson[1,2], HM Barham[1], EC Chinje[1], GE Adams[1], AL Harris[2] and IJ Stratford[1]

[1]*MRC Radiobiology Unit, Chilton, Didcot, Oxon OX11 ORD, UK;* [2]*ICRF Clinical Oncology Unit, University of Oxford, Churchill Hospital, Oxford, OX3 7LJ, UK.*

**Summary** P450 reductase (NADPH:cytochrome P450 reductase, EC 1.6.2.4) is known to be important in the reductive activation of the benzotriazene-di-*N*-oxide tirapazamine (SR 4233). Using a panel of six human breast adenocarcinoma cell lines we have examined the relationship between P450 reductase activity and sensitivity to tirapazamine. The toxicity of tirapazamine was found to correlate strongly with P450 reductase activity following an acute (3 h) exposure under hypoxic conditions, the drug being most toxic in the cell lines with the highest P450 reductase activity. A similar correlation was also observed following a chronic (96 h) exposure to the drug in air but not following acute (3 h) exposure in air. We have also determined the ability of lysates prepared from the cell lines to metabolise tirapazamine to its two-electron reduced product, SR 4317, under hypoxic conditions using NADPH as an electron donor. The rate of SR 4317 formation was found to correlate both with P450 reductase activity and with sensitivity to tirapazamine, the highest rates of SR 4317 formation being associated with the highest levels of P450 reductase activity and the greatest sensitivity to the drug. These findings indicate a major role for P450 reductase in determining the hypoxic toxicity of tirapazamine in breast tumour cell lines.

**Keywords:** P450 reductase; hypoxia; bioreductive drugs; tirapazamine; breast cancer

The presence of regions of low oxygen tension in a variety of human solid tumours is now well established (Gatenby *et al.*, 1988; Hockel *et al.*, 1991; Vaupel *et al.*, 1991; Lartigau *et al.*, 1992) and this hypoxia can predispose to failure of some treatments with radiotherapy (Gatenby *et al.*, 1988; Hockel *et al.*, 1993; Okunieff *et al.*, 1993). The resistance of hypoxic cells to radiation has long been recognised in experimental systems (Moulder and Rockwell, 1987; Rockwell and Moulder, 1990), and methods designed to overcome this resistance have included the development of hypoxic cell radiosensitisers (Adams, 1976; Stratford, 1992) and more recently the combination of nicotinamide and carbogen (Chaplin *et al.*, 1991). An alternative strategy has been to develop agents that selectively kill hypoxic cells (Adams and Stratford, 1986; Stratford *et al.*, 1986; Zeman *et al.*, 1986; Kennedy, 1987). This approach involves the exploitation of various biochemical processes that can result in selective reductive activation of drugs at low oxygen tensions (Kennedy, 1987; Workman, 1992; Workman and Stratford, 1993). In principle, this concept was first proposed in the context of quinone bioreductive drugs, with the notion that the low levels of oxygen in solid tumours could allow reductive activation of a drug to give a product which was more toxic than the parent compound (Lin *et al.*, 1972). Subsequently, agents have been identified that are substantially more toxic to hypoxic compared with oxic cells and this is the basis for their tumour selectivity (Adams and Stratford, 1986, 1994; Stratford and Stephens, 1989.) Classes of compound that are in, or are about to enter, clinical trial as bioreductive drugs include (1) RB6145, the lead compound from a series of dual-function, alkylating nitroimidazoles (Cole *et al.*, 1990, 1991, 1992; Jenkins *et al.*, 1990) (2) quinones such as the mitomycin C analogue porfiromycin (Rockwell *et al.*, 1988) and the indoloquinone EO9 (Hendriks *et al.*, 1993); and (3) the benzotriazene-di-*N*-oxide tirapazamine (SR4233, WIN-59075) (Brown and Lemmon, 1990; Brown, 1993).

As indicated above, the major rationale for the development of bioreductive drugs has been the presence of tumour hypoxia. However, for these agents to be effective they require metabolic activation, catalysed by the cellular complement of reductase enzymes. These can include isozymes of the P450 system, cytochrome P450 reductase, cytochrome $b_5$ reductase, xanthine oxidase etc (Walton *et al.*, 1989; Workman, 1992; Hodnick and Sartorelli, 1993). Therefore, it has been proposed that hypoxic cells could be more effectively targeted if differences in the levels of reductases in various cell types were taken into account in order to direct appropriate agents to particular human tumours based on their enzymology (Workman and Walton, 1986; Workman and Stratford, 1993; Workman, 1994).

The potential usefulness of the 'enzyme-directed' approach to bioreductive drug development has been very clearly demonstrated with the mitosense EO9. The toxicity of this agent in air is highly dependent on the cellular expression of the obligate two-electron reductase, DT-diaphorase, (Robertson *et al.*, 1992, 1994; Plumb *et al.*, 1994; Smitskamp-Wilms *et al.*, 1994). Under hypoxic conditions, reduction of EO9 can also be catalysed by cytochrome P450 reductase (Bailey *et al.*, 1994) an enzyme which has been shown to play an important role in the metabolism of 2-nitroimidazoles (Walton and Workman, 1987) and *N*-oxides such as tirapazamine (Walton *et al.*, 1989).

In previous studies on the metabolism of tirapazamine using mouse and rat liver microsomes (Walton *et al.*, 1989, 1992; Lloyd *et al.*, 1991) and tumour cell lysates (Wang *et al.*, 1993) it was shown that both cytochrome P450 and cytochrome P450 reductase contribute to the overall reduction of tirapazamine to its two-electron reduced product SR 4317. Cytochrome P450 is dependent upon the presence of cytochrome P450 reductase for its catalytic activity (Peterson and Prough, 1986). Hence, P450 reductase will play both direct and indirect roles in the reduction of tirapazamine. Moreover, tirapazamine is reduced by purified rat liver cytochrome P450 reductase (Walton *et al.*, 1989, Cahill and White, 1990) leading to the production of strand breaks in co-incubated plasmid DNA (Fitzsimmons *et al.*, 1994). Therefore, the aim of this work was to assess the likely importance of P450 reductase activity for determining the sensitivity of tumour cells to tirapazamine. To do this we have measured P450 reductase activity in a panel of six human breast adenocarcinoma cell lines, and compared this with their sensitivity to tirapazamine under hypoxic and aerobic conditions. Given the role of P450 reductase in reducing tirapazamine, we have also determined the ability of the different cell lines to metabolise tirapazamine to its reduced product SR 4317 under hypoxic conditions.

Correspondence: IJ Stratford
Received 2 May 1995; revised 22 June 1995; accepted 27 June 1995

### Materials and methods

### *Chemicals*

Tirapazamine, SR 4317 and SR 4330 were obtained from Sterling Winthrop Ltd or synthesised in house using previously described methods (Seng and Ley, 1972). NADPH was purchased from Boehringer Mannheim (Lewes, UK). HPLC-grade methanol was purchased from Merck (Lutterworth, UK). All other reagents were of analytical grade and were purchased from Sigma (Poole, UK). Tissue culture medium was obtained from ICRF (Clair Hall Labs, UK) and fetal calf serum from Sigma.

### *Cells and culture*

Table I lists the six human breast tumour cell lines used in this work. All cell lines were maintained in exponential growth phase in RPMI-1640 medium (except for SKBr-3 cells which were maintained in E4 medium), supplemented with 2 mM glutamine and 10% (v/v) fetal calf serum. MCF-7 (EP) cells are early passage cells (passage 60–70) from the original strain established by Soule *et al.* (1973). In one set of experiments, indicated by footnote d in Table I, a late passage strain of MCF-7 cells was used (Newcastle Strain, MCF-7 (LP), passage > 300). Measurements of P450 reductase activity were made in each strain and showed no significant difference.

### *Drug sensitivity*

Dose–response curves were determined using the MTT proliferation assay, which is based on the ability of viable cells to convert a soluble tetrazolium salt, MTT, into purple formazan crystals (Mossman, 1983). The optical density of the dissolved crystals is proportional to the number of viable cells, although this varies between cell lines as the conversion of MTT to formazan depends on the level of mitochondrial dehydrogenase activity in each cell line (Carmichael *et al.*, 1987). The conditions for carrying out the assay have been described elsewhere (Robertson *et al.*, 1994; Carmichael *et al.*, 1987) and require plating $1 \times 10^3 - 5 \times 10^3$ cells (depending on cell line) into each well of a 24-well glass dish 3 h before exposure to tirapazamine for 3 h at 37°C in either air or hypoxia. The cells were then washed free of drug and allowed to grow for 4 days in 0.4 ml of fresh medium. Alternatively, chronic (96 h) aerobic exposures were carried out in a 96-well-plate format. After 4 days, MTT was added (0.2 mg $ml^{-1}$ medium) and cells incubated for a further 4 h. Culture medium and unconverted MTT were removed and the formazan crystals dissolved in 0.2 ml dimethyl-sulphoxide (DMSO). An aliquot of 25 µl of glycerine buffer, pH 10.5 (Plumb *et al.*, 1989) was then added and the optical density at 540 nm measured on a multiwell spectrophotometer. Values of $IC_{50}$, the concentration of tirapazamine required to reduce optical density by 50% compared with the untreated controls, were used as the measure of cellular sensitivity to a given treatment. The $IC_{50}$ values quoted in Table I are the means of at least three independent experiments conducted on different days.

### *Cell lysates*

Cells in exponential growth phase were washed twice with phosphate-buffered saline (PBS) and harvested using a sterile cell scraper. Following centrifugation at 800 r.p.m. for 8 min, pellets were taken and washed in ice-cold hypotonic nuclear buffer A (10 mM Hepes/potassium hydroxide pH 7.4, 1.5 mM magnesium chloride, 10 mM potassium chloride, 0.05 mM DTT). Following repelleting, cells were suspended in 1.5 ml of nuclear buffer A and allowed to stand for 10 min at 4°C. Suspensions were sonicated using an MSE Soniprep 150 for 3 × 5 s at a nominal frequency of 23 kHz and an oscillation amplitude of between 5 and 10 µm. Samples were placed on ice between each sonication. The suspensions were allowed to stand on ice for a further 10 min, and then centrifuged at 7800 *g* for 15 min at 4°C. The resulting lysate was removed and stored in liquid nitrogen until required. The protein concentration of the cell lysates was determined using the Bio-Rad protein dye assay (Bradford, 1976) using high-grade bovine serum albumin (BSA) as the standard.

### *NADPH:P450 reductase activity*

P450 reductase activity was determined spectrophotometrically as the NADPH-dependent reduction of cytochrome *c*. Each incubation comprised 400 µl of the cytochrome *c* (final concentration 50 µM), 100 µl of 10 mM potassium cyanide acetonitrile (final concentration 1 mM) and 100–300 µg lysate protein (50–100 µl volume) made up to 0.98 ml with 100 mM phosphate buffer, pH 7.6. The reaction was equilibrated to 37°C and initiated by addition of 20 µl 10 mM NADPH to the test cuvette (final concentration 200 µM) and the rate of reduction of cytochrome *c* was monitored at 550 nm for 3 min against a blank without NADPH. Initial rates of reaction were based on an extinction coefficient of 21 $mM^{-1}$ $cm^{-1}$ calculated and expressed as nmol cytochrome *c* reduced per min per mg lysate protein.

### *Western blot analysis*

Samples of cells harvested for enzyme assays were washed in PBS buffer containing 1 mM phenylmethylsulphonyl fluoride, 1 mM benzamide, 50 µg $ml^{-1}$ leupeptin and 50 µg $ml^{-1}$ soyabean trypsin inhibitor. Cells were lysed in 1 ml 2% w/v sodium dodecyl sulphate (SDS), plus inhibitors in PBS at 65°C for 5 min, then suspensions passed up and down a fine-gauge needle in order to break up the DNA. Samples were stored at – 20°C. Proteins were subsequently resolved by 7.5% SDS-polyacrylamide gel electrophoresis, and proteins on the gel were electrophoretically transferred overnight to a nitrocellulose hybridisation transfer membrane. The membrane was washed with blocking buffer (20 mM Tris-HCL (pH 7.5), 0.9% sodium chloride, 0.5% Tween 20, 1% low fat Marvel), and incubated for 60 min with specific NADPH:P450 reductase rabbit antibody (dilution 1:500, supplied by Professor CR Wolf, University of Dundee, UK). After washing, horseradish peroxidase-conjugated goat anti-rabbit antibody (dilution 1:5000) was added and incubated for 30 min. All antibodies were diluted in blocking buffer. The membrane was developed using an enhanced chemiluminescence Western blotting detection kit (Amersham, UK).

### *Metabolism of tirapazamine by cell lysates*

Incubations were carried out under nitrogen at 37°C in 4 ml amber glass vials (Chromacol, Welwyn Garden City, UK) sealed with Subaseal (Aldrich, Gillingham, UK). The 500 µl incubation volume comprised 100 µl of cell lysate (maximum final protein concentration of 1.5 mg $ml^{-1}$), 100 µl of NADPH (5 mM dissolved in incubation buffer, giving a final incubation concentration of 1 mM), 20 µl of tirapazamine (50 mM dissolved in DMSO to give a final incubation concentration of 2 mM) and 280 µl of incubation buffer (0.2 M potassium phosphate buffer, pH 7.4). After preincubation under nitrogen for 10 min, the reaction was started by addition of tirapazamine using a Hamilton syringe inserted through the Subaseal. Reactions were stopped after 40 min by transferring 2 × 200 µl aliquots of the incubate into polypropylene vials containing 50 µl of internal standard [4-nitroquinoline *N*-oxide; 0.4 mg $ml^{-1}$ in 20% (v/v) ethanol] and 400 µl of methanol. Samples were vortexed vigorously for 2 min, centrifuged for 5 min at 3000 r.p.m. and 200 µl aliquots of the supernatants injected onto the HPLC system for analysis. Three lysate preparations for each cell line were incubated, each in duplicate, with duplicate analyses. Formation of SR 4317 was linear for at least 40 min and up to a protein concentration of 1.5 mg $ml^{-1}$, determined using

SkBr3 lysates, which have the highest P450 reductase activity amongst the panel of cell lines.

*HPLC*

Concentrations of SR 4317 and SR 4330 in incubation samples were determined by isocratic reverse phase HPLC (Walton and Workman, 1990). Chromatography was performed using a Waters μBondapak phenyl 4 μm radial compression cartridge in a Waters radial compression module (Waters Chromatography, Watford, UK) and protected with a phenyl guard column. The mobile phase consisted of 32% methanol in water delivered at a flow rate of 3 ml $min^{-1}$. Detection was at 267 nm. Approximate retention times under these conditions were 2.7, 4.6, 5.2 and 8.4 min for tirapazamine, SR 4330, SR 4317 and 4-nitroquinoline *N*-oxide (internal standard) respectively. Concentrations of metabolites were calculated from peak height ratios and comparison with calibration curves (0–500 μM) prepared by spiking lysate preparations with known amounts of metabolite.

*Statistical analysis*

The data were analysed using the standard model for a linear functional relationship with sampling errors in both variables. The data were logarithmically transformed and the pooled variance of each data set was calculated. It is assumed that the random sampling errors are normally and independently distributed with zero means and variances inversely proportional to the sample size. For statistical analysis of any two data sets, the model is fitted to the observations by the method of weighted least squares, each sample mean being weighted in direct proportion to the sample size. The statistical goodness-of-fit for any two data sets was tested by calculating the weighted mean-square deviation of the observations for mean $x_n$ and mean $y_n$ from the fitted model, and comparing this mean-square with the pooled variance within samples by a variance–ratio test. The statistical significance of the estimate of the slope of the straight line of best fit was tested by a Student's *t*-test.

### Results

The toxic effect of tirapazamine on the breast cancer cell lines was determined in two sets of experiments. Firstly, by exposing cells to drug for 3 h under aerobic or hypoxic conditions and secondly, by growing cells in air for 96 h in the presence of tirapazamine. Cytotoxicity was measured by the MTT assay and typical survival curves, derived from individual experiments are given in Figure 1. The data illustrate the large increase in toxicity that occurs when drug exposure is under hypoxic compared with aerobic conditions and also show that the absolute potency of the drug in air and hypoxia can vary between cell lines. From such curves values of $IC_{50}$ can be obtained. Mean values derived from at least six independent, replicate experiments which update our previously published data (Patterson *et al.*, 1994) are given in Table I. (Cell lines are listed in order of sensitivity to tirapazamine under hypoxic conditions). Also included in the table are values of differential toxicity. This is the ratio of $IC_{50}$ values obtained following 3 h exposure to tirapazamine in air *vs* hypoxia. Each cell line is more sensitive to tirapazamine under hypoxia, with values of differential toxicity covering a 5- to 6-fold range from 13 to 69. There is no relationship between the value of differential toxicity and drug potency under either aerobic or hypoxic conditions.

The level of NADPH-dependent P450 reductase activity, measured in lysates from up to four separate cultures of each cell line, is given in Table I. The enzyme activity in the breast cancer cell lines covers a 6-fold range. This contrasts with the activity of the reductase DT-diaphorase in these cell lines, which varies by a factor of 260, the highest expressor being ZR-75 and the lowest MDA-231 cells (Robertson *et al.*, 1994). We have shown previously that the toxicity of tirapazamine in breast and lung cancer cells following a 96 h aerobic exposure does not depend on DT-diaphorase activity (Patterson *et al.*, 1994). However, as can be seen in Figure 2, there is a clear relationship between P450 reductase activity and tirapazamine toxicity in breast cancer cells following chronic (96 h) exposure in air. The highest toxicity (lowest value of $IC_{50}$) occurs in the cell line with highest activity of P450 reductase (SKBr3). The value of slope derived from these data is – 1.6 ± 0.3 ($P = 0.006$).

The importance of P450 reductase in the activation and toxicity of tirapazamine in the breast cancer cells has been characterised further by carrying out acute (3 h) exposure to drug under aerobic and hypoxic conditions. The dependence of $IC_{50}$ on P450 reductase activity is shown in Figure 3. Under hypoxic conditions there is a highly significant relationship between intracellular enzyme activity and drug tox-

**Figure 1** Examples of individual survival curves derived by the MTT assay for SKBr3 cells (circles) and ZR-75 cells (squares) exposed to tirapazamine for 3 h under aerobic (closed symbols) or hypoxic (open symbols) conditions.

**Table I** Human breast cancer cell lines: response to tirapazamine under aerobic or hypoxic conditions, activity of NADPH-dependent P450 reductase and ability to metabolise tirapazamine to the two-electron reduced product SR 4317 under hypoxic conditions

| Cell line | $IC_{50}$ μM[a] Aerobic exposure (96 h) | Aerobic exposure (3 h) | Hypoxic exposure (3 h) | Differential toxicity[b] | NADPH:P450 reductase activity nmol substrate reduced $min^{-1}$ $mg^{-1}$ lysate protein | SR 4317 formation velocity | Western blot densitometry values (arbitrary units)[c] |
|---|---|---|---|---|---|---|---|
| SKBr 3 | 10.4 ± 0.52 | 167.2 ± 48.2 | 5.1 ± 2.0 | 32.8 | 39.8 ± 3.2 | 22.4 ± 3.4 | 3187 ± 495 |
| MDA-468 | 18.1 ± 7.0 | 180.9 ± 76.6 | 7.3 ± 2.1 | 24.9 | 20.5 ± 1.8 | 11.0 ± 1.9 | 1372 ± 251 |
| T47D | 20.9 ± 5.3 | 301.3 ± 67.8 | 12.7 ± 2.0 | 23.7 | 16.1 ± 3.2 | 13.2 ± 2.7 | 1081 ± 253 |
| MCF-7 (EP) | 24.7 ± 9.9[d] | 546.0 ± 61.3 | 15.0 ± 3.9 | 36.4 | 16.1 ± 1.2 | 10.2 ± 0.9 | 960 ± 136 |
| ZR-75-1 | 26.6 ± 3.6 | 1295 ± 502 | 18.9 ± 2.4 | 68.5 | 10.8 ± 1.8 | 9.1 ± 1.4 | 473 ± 120 |
| MDA-231 | 35.4 ± 4.1 | 307.8 ± 70.4 | 23.8 ± 4.0 | 12.9 | 6.9 ± 1.8 | 3.7 ± 0.7 | 231 ± 56 |

[a]Values ± s.d. updated from Patterson *et al.* (1994) with data being derived from up to five additional experiments on each cell line. [b]Ratio of $IC_{50}$ values for 3 h drug exposures under aerobic *vs* hypoxic conditions. [c]Data derived from Western blots of three separate lysates of each cell line. [d]Data from MCF-7 (LP), NADPH:P450 reductase activity = 18.7 ± 3.4 nmol cytochrome *c* reduced $min^{-1}$ $mg^{-1}$ protein.

**Figure 2** Dependence of $IC_{50}$ values, derived by MTT assay of human breast cancer cells following 96 h exposure to tirapazamine in air, on NADPH cytochrome P450 reductase activity. Bars indicate standard errors.

**Figure 3** Dependence of $IC_{50}$ values of human breast cancer cells exposed to tirapazamine for 3 h under aerobic (●) or hypoxic (○) conditions on NADPH cytochrome P450 reductase activity. Bars indicate standard errors.

icity (slope value = − 1.10 ± 0.12; $P < 10^{-10}$). In contrast, aerobic toxicity at 3 h does not show such a dependency (slope value = − 1.34 ± 0.84; $P = 0.19$).

Further evidence for a relationship between P450 reductase activity and acute hypoxic drug toxicity is provided by a Western blotting experiment in which the levels of P450 reductase protein in the cell lines were measured using a specific NADPH-dependent P450 reductase antibody. This is shown in Figure 4. An equivalent quantity of protein from each cell line was loaded onto the gel in lanes from left to right in rank order of their sensitivity to tirapazamine. It is apparent that the levels of P450 reductase protein decrease from left to right, i.e. the lowest level of protein occurring in the cell line with the greatest resistance to tirapazamine under hypoxic conditions. Values of the densitometry readings from these Western blots are given in Table I. When compared with values of enzyme activity in each cell line there is a strong correlation (slope ratio = 2.43 ± 0.32, $P < 10^{-5}$).

P450 reductase has previously been shown to play an important role in the metabolism of tirapazamine (Walton *et al.*, 1989, 1992; Lloyd *et al.*, 1991; Wang *et al.*, 1993). Furthermore, the rate of metabolism has been linked to cytotoxic efficiency under anaerobic conditions (Biedermann *et al.*, 1991). In order to determine whether P450 reductase is a major factor contributing to the metabolism of tirapazamine in breast cancer cells, we have measured the rate at which cell lysates reduce tirapazamine to its two-electron reduced product, SR 4317 under hypoxic conditions. The structures of these compounds are given in Figure 5. No formation of the deoxygenated four-electron reduced product, SR 4330, was detected in these experiments. These rates of formation of SR 4317 are given in Table I and a plot of P450 reductase

**Figure 4** Western immunoblot of the relative NADPH cytochrome P450 reductase expression in breast cancer cells ranked in order of *in vitro* hypoxia sensitivity to tirapazamine. Gels were loaded with equal protein concentrations of lysates from each cell line. Microsomes derived from livers in phenobarbital-treated mice were used as a positive control (67 μg of protein in 100 μl). Phenobarbital was given at 80 mg kg$^{-1}$ i.p. four times daily and mice sacrificed 24 h after the last injection (Walton *et al.*, 1992).

**Figure 5** Structure of tirapazamine (SR 4233) and its two-electron reduced product SR 4317.

**Figure 6** Dependence on NADPH cytochrome P450 reductase activity for the ability of breast cell lysates to convert tirapazamine to SR 4317 under hypoxic conditions. Bars indicate standard errors.

activity in the breast cancer cell lines *vs* the rate of SR 4317 formation catalysed by lysates of each cell line, with NADPH provided as the electron source, is given in Figure 6. It can be seen from these data that there is a strong correlation (slope value = 1.10 ± 0.23; $P = 0.009$) between P450 reductase activity and SR 4317 formation, with higher values of enzyme activity resulting in greater rates of metabolism. When similar experiments were carried out in air, no metabolism was detected.

### Discussion

Previous studies have shown that tirapazamine is reductively metabolised to SR 4317 by purified rat liver NADPH:

cytochrome P450 reductase (Cahill and White, 1990; Fitzsimmons *et al.*, 1994). Lloyd *et al.* (1991) identified a free radical intermediate formed during microsomal reduction of tirapazamine and demonstrated, following the use of appropriate inhibitors, that P450 reductase was the enzyme responsible for the radical production. In the present work it is demonstrated that NADPH: cytochrome P450 reductase, present in human breast cancer cell lines *in vitro*, plays a major role in the reduction of tirapazamine and thus in determining the toxicity of the drug. The relationship between P450 reductase activity and drug toxicity is apparent following exposure of cells to tirapazamine under hypoxic conditions (3 h) and following 96 h exposure in air. However, such a dependence is not apparent after only 3 h treatment in air. These results can be interpreted on the basis of the activation toxicity scheme outlined below.

In the presence of P450 reductase, tirapazamine (SR 4233) will be subject to one-electron reduction to give the radical anion (SR 4233$^-$), which in its protonated form has been identified as a nitroxide radical (Lloyd *et al.*, 1991). In the absence of oxygen the radical will either disproportionate giving tirapazamine and SR 4317 (which can be regarded as a detoxification reaction) or undergo reaction with biomolecules e.g. hydrogen atom abstraction from DNA (Baker *et al.*, 1988). The latter reaction could yield a DNA radical and SR 4317. There is some evidence to suggest that this latter process is the first step in a type of chain reaction that could result in multiple damaged sites from a single reductive event. Laderoute *et al.* (1988) demonstrated the existence of a short chain reaction during the radiolytic reduction of tirapazamine in a solution containing formate. P Wardman (personal communication) has suggested that SR 4233$^-$-mediated hydrogen atom abstraction from the sugar backbone of DNA will result in strand breakage and the formation of a sugar radical and this radical can subsequently reduce another molecule of tirapazamine etc., thereby amplifying the damage to DNA. It has been suggested that such multiply damaged sites in DNA (which could be produced by the mechanism described by Wardman) could be the critical lesions for tirapazamine toxicity (Brown, 1993). Therefore, if the assumption is made that the breast cancer cell lines have similar abilities to repair damage caused by SR 4233$^-$, then the level of damage under hypoxic conditions will be dependent upon the rate at which SR 4233$^-$ is formed, i.e. dependent on the cellular activity of P450 reductase. Incidental support for this comes from observations of Keohane *et al.* (1990) who showed that tirapazamine was less toxic towards a CHO cell line, designated $MMC^r$, which is deficient in P450 reductase activity compared with the parental line from which it was derived (CHO-K1).

A correlation between P450 reductase activity and cellular sensitivity to tirapazamine is observed in air following chronic (96 h) but not acute (3 h) exposure to the drug. This difference and the difference between the values of $IC_{50}$ for acute hypoxic *vs* aerobic exposure (values of differential toxicity varying from 12.9 to 68.5) are most likely to be explained by the effects of redox cycling which will occur in air. During this process SR 4233$^-$ will be formed following one-electron reduction of tirapazamine, catalysed by P450 reductase. In the presence of oxygen the reduced product will be back-oxidised to tirapazamine, with the concomitant production of oxygen radicals. Thus, in air, cellular damage may be mediated by both superoxide radicals and SR 4233$^-$. Relatively high concentrations of tirapazamine were used during the acute exposure to air, which is likely to result in the formation of sufficiently high concentrations of oxygen radicals to overwhelm cellular antioxidant defence mechanisms. Thus, the variation in cellular sensitivity to tirapazamine following acute exposure in air may well reflect variations in the tolerance of the different cell types both to oxidative damage and to damage by SR 4233$^-$, thereby explaining the apparent lack of a relationship between the drug toxicity and P450 reductase activity under these conditions. Evidence suggesting that the type of damage occurring in cells following acute (3 h) exposure to tirapazamine in either air or nitrogen can be different, comes from studies comparing drug toxicity in a V79 cell line and a DNA repair-deficient mutant line derived from it (Keohane *et al.*, 1990). V79 and irs-1 cells have similar sensitivities to tirapazamine in hypoxia whereas, in air irs- 1 cells are 10-fold more sensitive (as measured by values of $IC_{90}$). The irs-1 cells are radiation sensitive and are defective in the fidelity of DNA strand break repair. If it is assumed that the two cell lines have a similar ability to metabolise tirapazamine i.e., similar P450 reductase activities, then the difference in sensitivities between V79 and irs-1 cells observed in air but not in nitrogen would reflect differences in the damage caused under these conditions. That is, in hypoxia, the damage is caused by P450 reductase-mediated formation of tirapazamine radicals which can interact with DNA, whereas, following acute exposures in air, cells are additionally damaged by oxygen radicals. Support for the latter is indicated by the finding that the addition of metal chelators can protect cells against the toxic effects of tirapazamine in air but not in nitrogen (Herscher *et al.*, 1994).

Following chronic (96 h) exposure of cells to tirapazamine in air, a relationship between P450 reductase activity and drug toxicity is observed. However, the concentrations of drug used in these experiments were 10- to 50-fold lower than those used in the acute (3 h) aerobic experiments. Therefore, it might be expected that the concentrations of oxygen radicals formed by redox cycling are likely to be low enough to be accommodated by the cellular antioxidant defence mechanisms. Thus, for this treatment condition, toxicity will be due predominantly to damage caused by SR 4233$^-$.

Lysates derived from the breast cancer cells have been assayed for their ability to metabolise tirapazamine to SR 4317 under hypoxic conditions and this is shown to depend on P450 reductase activity (Figure 5). Since conversion of tirapazamine to its one-electron reduced product (SR 4233$^-$) is enzyme mediated and, under hypoxic conditions, subsequent conversion to SR 4317 is a chemical process, we believe that measurement of formation of SR 4317 is an indirect measure of 'toxic' radical formation. Thus, it would be expected that formation of SR 4317 by cell lysates should correlate with the $IC_{50}$ for tirapazamine toxicity under hypoxic conditions, which indeed is the case (slope value $= -0.93 \pm 0.30$, $P = 0.036$). Further, this correlation strongly suggests that conversion of tirapazamine to SR 4317 by direct two-electron reduction, e.g. by DT-diaphorase (which will bypass the one-electron reduction product), does not contribute to toxicity (Patterson *et al.*, 1994).

The level of hypoxia (Koch, 1993), the ability of cells to repair DNA damage (Keohane *et al.*, 1990; Biedermann *et al.*, 1991) and the level of cellular reductases, will all influence the therapeutic selectivity of tirapazamine. However, in breast cancer cells the activity of NADPH:cytochrome P450 reductase is important for the hypoxic toxicity of tirapazamine. In the application of these observations it is useful to note that the expression of P450 reductase protein (measured by immunoblot analysis) correlates well with the activity of P450 reductase, indicating that the P450 reductase protein is catalytically active. Thus, enzyme profiling of cells and tissues, together with a measure of tumour hypoxia, may provide a useful screen for predicting the activity of tirapazamine *in vivo* (Philip *et al.*, 1994; Rampling *et al.*, 1994). The work of Wang *et al.* (1993) and Biedermann *et al.*

(1991) has shown that the hypoxic toxicity of tirapazamine correlates with the overall rate of metabolism of the parent drug. In a mouse and a human sarcoma cell line, DT-diaphorase and cytochromes P450 were shown to contribute to the metabolism of tirapazamine under hypoxic conditions but no evidence was provided for the role of P450 reductase in these cells (Wang et al., 1993). Our previous studies on DT-diaphorase clearly indicate that this enzyme does not play an important role in determining sensitivity to tirapazamine in air or hypoxia in the breast cancer cells (Patterson et al., 1994). The role of P450 isozymes is less clear, particularly since cells in culture lose their ability to regulate the expression of P450 genes (Paine, 1990). From studies using chemical and antibody inhibitors in enzyme-induced mouse liver microsomes, it has been suggested that Cyp2b and Cyp2c can contribute to tirapazamine reduction (Riley et al., 1993). However, this has yet to be linked to toxicity. In clinical samples of breast tumour tissue only the presence of CYP1A has been demonstrated unequivocally (Murray et al., 1991), whereas in breast xenografts in mice CYPs 2A, 2B, 2C, 3A and 4A have been detected, but only to a significant extent after enzyme induction (Smith et al., 1993).

In conclusion, we have demonstrated that NADPH:P450 reductase plays a major role in determining the hypoxic toxicity of tirapazamine in breast cancer cells. This provides an example of the enzyme-directed approach to bioreductive drug development, and we are currently evaluating the generality of this approach for tirapazamine in other tumour types.

**Acknowledgements**
We thank Professor J Carmichael, Drs S Houlbrook and D Talbot and Ms N Robertson for their constructive advice during the course of this work. Mr. John Nolan is thanked for synthesis of tirapazamine, SR 4317 and SR 4330, and Professor CR Wolf for supply of P450 reductase antibody. We are also grateful to Mr David Papworth, who carried out the statistical analysis, and Ms J McCourt, who prepared the manuscript. This study was supported in part by NCI Grant No. P01-CA-55165 (HMB and ECC) and Sterling Winthrop (AVP).

**References**

ADAMS GE. (1976). Hypoxic cell sensitizers for radiotherapy. In *Cancer, a Comprehensive Treatise*, Vol. 6, *Radiotherapy, Surgery and Immunotherapy*. Becker FF. (ed.) pp. 181–223. Plenum Press, New York.

ADAMS GE AND STRATFORD IJ. (1986). Hypoxia-mediated nitro-heterocyclic drugs in the radio and chemotherapy of cancer: An overview. *Biochem. Pharmacol.*, **35**, 71–76.

ADAMS GE AND STRATFORD IJ. (1994). Bioreductive drugs for cancer therapy: The search for tumour specificity. *Int. J. Radiat. Oncol. Biol. Phys.*, **29**, 231–238.

BAILEY SM, LEWIS AD AND WORKMAN P. (1994). Involvement of NADPH: Cytochrome P450 reductase in activation of EO9 to a DNA damaging species. *Br. J. Cancer*, **69**, (suppl. XXI), 57.

BAKER MA, ZEMAN EM, HIRST VK AND BROWN JM. (1988). Metabolism of SR 4233 by Chinese Hamster ovary cells: Basis of selective hypoxic cytotoxicity. *Cancer Res.*, **48**, 5947–5952.

BIEDERMANN KA, WANG J, GRAHAM RP AND BROWN JM. (1991). SR 4233 cytotoxicity and metabolism in DNA repair-competent and repair-deficient cell cultures. *Br. J. Cancer*, **63**, 358–362.

BROWN JM. (1993). SR 4233 (Tirapazamine): a new anticancer drug exploiting hypoxia in solid tumours. *Br. J. Cancer*, **67**, 1163–1170.

BROWN JM AND LEMMON MJ. (1990). Potentation by the hypoxic cytotoxin SR 4233 of cell killing produced by fractionated irradiation of mouse tumours. *Cancer Res.*, **50**, 7745–7749.

BRADFORD M. (1976). A rapid and sensitive method for quantification of microgram quantities of protein utilising the principle of protein dye binding. *Anal. Biochem.*, **72**, 248–254.

CAHILL A AND WHITE INH. (1990). Reductive metabolism of 3-amino- 1,2,4-benzotriazine-1,4-dioxide (SR 4233) and the induction of unscheduled DNA synthesis in rat and human derived cell lines. *Carcinogenesis*, **11**, 1407–1411.

CARMICHAEL J, DE GRAFF WG, GAZDAR AF, MINNA JD AND MITCHELL JB. (1987). Evaluation of a tetrazolium-based semi-automated colorimetric assay: Assessment of chemosensitivity testing. *Cancer Res.*, **47**, 936–941.

CHAPLIN DJ, HORSMAN MR AND AOKI DS. (1991). Nicotinamide, Fluosol DNA and Carbogen: a strategy to reoxygenate acutely and chronically hypoxic cells *in vivo*. *Br. J. Cancer*, **63**, 109–113.

COLE S, STRATFORD IJ, ADAMS GE, FIELDEN EM AND JENKINS TC. (1990). Dual function 2-nitroimidazoles and hypoxic cell radiosensitizers and bioreductive cytotoxins: *in vivo* evaluation in KHT murine sarcomas. *Radiat. Res.*, **124**, S38–S43.

COLE S, STRATFORD IJ, BOWLER J, NOLAN J, WRIGHT EG, LORIMORE SA AND ADAMS GE. (1991). Oral (po) dosing with RSU 1069 or RB 6145 maintains their potency as hypoxic cell radiosensitizers and cytotoxins but reduces systemic toxicity compared with parenteral (ip) administration of mice. *Int. J. Radiat. Oncol. Biol. Phys.*, **21**, 387–395.

COLE S, STRATFORD IJ, FIELDEN EM, ADAMS GE, LEOPOLD W, ELLIOT W, SUTO M AND SEBOLT-LEOPOLD J. (1992). Dual function nitroimidazoles less toxic than RSU 1069: Selection of candidate drugs for clinical trial (RB 6145 and/or PD 130908). *Int. J. Radiat. Oncol. Biol. Phys.*, **22**, 545–548.

FITZSIMMONS SA, LEWIS AD, RILEY RJ AND WORKMAN P. (1994). Reduction of 3-amino-1,2,4-benzotriazine-1,4-di-N-oxide (tirapazamine, WIN 59075, SR 4233) to a DNA-damaging species: a direct role for NADPH: cytochrome P450 oxidoreductase. *Carcinogenesis*, **15**, 1503–1510.

GATENBY RA, KESSLER HB, ROSENBLUM JS, COIA LR, MOLDOFSKY PJ, HARTZ WH AND BRODER GJ. (1988). Oxygen distribution in squamous cell carcinoma metastases and its relationship to the outcome of radiation therapy. *Int. J. Radiat. Oncol. Biol. Phys.*, **14**, 831–838.

HENDRIKS, HR, PIZAO PE, BERGER DP, KOOISTRA KL, BIBBY MC, BOVEN E, DREEF-VAN DER MEULEN HC, HENRAR REC, FIEBIG HH, DOUBLE JA, HORNSTRA HW, PINEDO HM, WORKMAN P AND SCHWARTSMANN G. (1993). EO9: A novel bioreductive alkylating indoloquinone with preferential solid tumour activity and lack of bone marrow toxicity in preclinical models. *Eur. J. Cancer*, **29A**, 897–906.

HERSCHER LL, KRISHNA MC, COOK JA, COLEMAN CN, BIAGLOW JE, TUTTLE SW, GONZALEZ FJ AND MITCHELL JB. (1994). Protection against SR 4233 (Tirapazamine) aerobic cytotoxicity by the metal chelators desferrioxamine and tiron. *Int. J. Radiat. Oncol. Biol. Phys.*, **30**, 879–885.

HOCKEL M, SCHLENGER K, KNOOP C AND VAUPEL P. (1991). Oxygenation of carcinomas of the uterine cervix: evaluation by computerised $O_2$ tension measurements. *Cancer Res.*, **51**, 6098–6102.

HOCKEL M, KNOOP C, SCHLENGER K, VORNDRAN B, BAUSSMAN E, MITZE M, KNAPSTEIN PG AND VAUPEL P. (1993). Intratumoral $pO_2$ predicts survival in advanced cancer of the uterine cervix. *Radiother. Oncol.*, **26**, 45–50.

HODNICK WF AND SARTORELLI AC. (1993). Reductive activation of mitomycin C by NADH: cytochrome $b_5$ reductase. *Cancer Res.*, **53**, 4907–4912.

JENKINS TC, NAYLOR MA, O'NEILL P, THREADGILL MD, COLE S, STRATFORD IJ, ADAMS GE, FIELDEN EM, SUTO MJ AND STIER MA. (1990). Synthesis and evaluation of 1-3-(2-haloethylamino)-propyl-2-nitroimidazoles as pro-drugs of RSU 1069 and its analogues, which are radiosensitizers and bioreductively activated cytotoxins. *J. Med. Chem.*, **33**, 2603–2610.

KENNEDY KA. (1987). Hypoxic cells as specific drug targets for chemotherapy. *Anticancer Drug Des.*, **2**, 181–194.

KEOHANE A, GODDEN J, STRATFORD IJ AND ADAMS GE. (1990). The effects of three bioreductive drugs (mitomycin C, RSU-1069 and SR 4233) on cell lines selected for their sensitivity to mitomycin C or ionising radiation. *Br. J. Cancer*, **61**, 722–726.

KOCH CJ. (1993). Unusual oxygen concentration dependence of toxicity of SR-4233, a hypoxic cell toxin. *Cancer Res.*, **53**, 3992–3997.

LADEROUTE K, WARDMAN P AND RAUTH AM. (1988). Molecular mechanisms for the hypoxia-dependent activation of 3-amino-1,2,4-benzotriazine-1,4-Dioxide (SR 4233). *Biochem. Pharmacol.*, **37**, 1487–1495.

LARTIGAU E, MARTIN L, LAMBIN P, HEIE-MEDER C, GERBAULET A, ESCHWEGE F AND GUICHARD M. (1992). Mesure de la pression partielle en oxygène dans des tumeurs du col utérin. *Bull. Cancer/Radiotherapie*, **79**, 199–206.

LIN AJ, COSBY LA, SHANSKY CW AND SARTORELLI AC. (1972). Potential bioreductive alkylating agents. I. Benzoquinone derivatives. *J. Med. Chem.*, **15**, 1247–1252.

LLOYD RV, DULING DR, RUMYANTSEVA GV, MASON RP AND BRIDSON PK. (1991). Microsomal reduction of 3-amino-1,2,4-benzotriazine 1,4-dioxide to a free radical. *Mol. Pharmacol.*, **40**, 440–445.

MOSSMAN T. (1983). Rapid colorimetric assay for cellular growth and survival: Application to proliferation and cytotoxicity assays. *J. Immunol. Methods*, **65**, 55–61.

MOULDER JE AND ROCKWELL S. (1987). Tumor hypoxia: its impact on cancer therapy. *Cancer Metast. Rev.*, **5**, 313–341.

MURRAY GI, FOSTER CO, BARNES TS, WEAVER RJ, EWEN SWB, MELVIN WT AND BURKE MD. (1991). Expression of cytochrome P450IA in breast cancer. *Br. J. Cancer*, **63**, 1021–1023.

OKUNIEFF P, HOECKEL M, DUNPHY EP, SCHLENGER K, KNOOP C AND VAUPEL P. (1993). Oxygen tension distributions are sufficient to explain the local response of human breast tumours treated with radiation alone. *Int. J. Radiat. Oncol. Biol. Phys.*, **26**, 631–636.

PAINE AJ. (1990). The maintainance of cytochrome P-450 in rat hepatocyte culture: some applications of liver cell cultures in the study of drug metabolism, toxicity and induction of the P-450 system. *Chem. Biol. Interact.*, **74**, 1–31.

PATTERSON AV, ROBERTSON N, HOULBROOK S, STEPHENS MA, ADAMS GE, HARRIS AL, STRATFORD IJ AND CARMICHAEL J. (1994). The role of DT-diaphorase in determining the sensitivity of human tumour cells to tirapazamine (SR 4233). *Int. J. Radiat. Oncol. Biol. Phys.*, **29**, 369–372.

PETERSON AJ AND PROUGH RA. (1986). Cytochrome P450 reductase and cytochrome b5 in cytochrome P450 catalysis. In *Cytochrome P450: Structure, Mechanism and Biochemistry*, Oritz de Montellano, RP. (ed.) pp. 89–119. Plenum Press: New York.

PHILIP AP, KAKLAMANIS L, RYLEY N, STRATFORD IJ, WOLF R, HARRIS A AND CARMICHAEL J. (1994). Expression of xenobiotic-metabolizing enzymes by primary and secondary hepatic tumours in man. *Int. J. Radiat. Oncol. Biol. Phys.*, **29**, 277–283.

PLUMB JA, MILROY R, AND KAYE SB. (1989). Effects of the pH dependence of 3-(4,5-dimethylthiazol-2-yl)-2,5-diphenyltetrazolium bromide-formazan absorption on chemosensitivity determined by a novel tetrazolium-based assay. *Cancer Res.*, **49**, 4435–4440.

PLUMB JA, GERRITSEN M, MILROY R, THOMSON P AND WORKMAN P. (1994). Relative importance of DT-diaphorase and hypoxia in the bioactivation of EO9 by human lung tumour cell lines. *Int. J. Radiat. Oncol. Biol. Phys.*, **29**, 295–299.

RAMPLING R, CRUICKSHANK G, LEWIS AD, FITZSIMMONS SA AND WORKMAN P. (1994). Direct measurement of $pO_2$ distribution and bioreductive enzymes in human malignant brain tumours. *Int. J. Radiat. Oncol. Biol. Phys.*, **29**, 427–431.

RILEY JR, HEMINGWAY SA, GRAHAM MA AND WORKMAN P. (1993). Initial characterisation of the major mouse cytochrome P450 enzymes involved in the reductive metabolism of the hypoxic cytotoxin 3-amino-1,2,4-benzotriazine-1,4-DI-N-Oxide (Tirapazamine, SR 4233, WIN 59075). *Biochem. Pharmacol.*, **45**, 1065–1077.

ROBERTSON N, STRATFORD IJ, HOULBROOK S, CARMICHAEL J AND ADAMS GE. (1992). The sensitivity of human tumour cells to quinone bioreductive drugs: what role for DT-diaphorase? *Biochem. Pharmacol.*, **44**, 409–412.

ROBERTSON N, HAIGH N, ADAMS GE AND STRATFORD IJ. (1994). Factors affecting sensitivity to EO9 in rodent and human tumour cells *in vitro*: DT-diaphorase activity and hypoxia. *Eur. J. Cancer*, **30A**, 1013–1019.

ROCKWELL S AND MOULDER JE. (1990). Hypoxic fractions of human tumours xenografted into mice: a review. *Int. J. Radiat. Onc. Biol. Phys.*, **19**, 197–202.

ROCKWELL S, KEYES SR AND SARTORELLI AC. (1988). Preclinical studies of porfiromycin as an adjunct to radiotherapy. *Rad. Res.*, **116**, 100–113.

SENG F AND LEY K. (1972). Simple synthesis of 3-amino-1,2,4 benzotriazine 1,4-dioxide. *Angew. Chem. Int.*, **XI**, 1009–1010.

SMITH G, HARRISON DJ, EAST N, RAE F, WOLF H AND WOLF CR. (1993). Regulation of cytochrome P450 gene expression in human colon and breast tumour xenografts. *Br. J. Cancer*, **68**, 57–63.

SMITSKAMP-WILMS E, PETERS GJ, PINEDO HM, VAN ARK-OTTE J AND GIACCONE G. (1994). Chemosensitivity to the indoloquinone EO9 is correlated with DT-diaphorase activity and its gene expression. *Biochem. Pharmacol*, **47**, 1325–1332.

SOULE D, VAZQUEZ J, LONG A, ALBERT S AND BRENNAN M. (1973). A human cell line from a pleural effusion derived from a breast carcinoma. *J. Natl. Cancer Inst.*, **51**, 1409–1413.

STRATFORD IJ. (1992). Concepts and developments in radiosensitization of mammalian cells. *Int. J. Radiat. Oncol. Biol. Phys.*, **22**, 529–532.

STRATFORD IJ AND STEPHENS MA. (1989). The differential hypoxic cytotoxicity of bioreductive agents determined *in vitro* by the MTT assay. *Int. J. Radiat. Oncol. Biol. Phys.*, **16**, 973–976.

STRATFORD IJ, O'NEILL P, SHELDON PW, SILVER ARJ, WALLING JM AND ADAMS GE. (1986). RSU 1069. A nitroimidazole containing an aziridine group – bioreduction greatly increases cytotoxicity under hypoxic conditions. *Biochem. Pharmacol.*, **35**, 105–109.

VAUPEL P, SCHLENGER K, KNOOP C AND HOCKEL M. (1991). Oxygenation of human tumours: evaluation of tissue oxygen distribution in breast cancers by computerised $O_2$ tension measurements. *Cancer Res.*, **51**, 3316–3322.

WALTON MI AND WORKMAN P. (1987). Nitroimidazole bioreductive metabolism: quantitation and characterisation of mouse tissue benznidazole nitroreductases *in vivo* and *in vitro*. *Biochem. Pharmacol.*, **36**, 887–896.

WALTON MI AND WORKMAN P. (1990). Enzymology of the reductive activation of SR4233: A novel benzotriazene di-N-oxide hypoxic cell cytotoxin. *Biochem. Pharmacol.*, **39**, 1735–1742.

WALTON MI, WOLF CR AND WORKMAN P. (1989). Molecular enzymology of the reductive bioactivation of hypoxic cell cytotoxins. *Int. J. Radiat. Oncol. Biol. Phys.*, **16**, 983–986.

WALTON MI, WOLF CR AND WORKMAN P. (1992). The role of cytochrome P450 and cytochrome P450 reductase in the reductive bioactivation of the novel benzotriazine DI-N-Oxide hypoxic cytotoxin 3-amino-1,2,4-benzotriazine-1,4-dioxide (SR 4233, WIN 59075) by mouse liver. *Biochem. Pharmacol.*, **44**, 251–259.

WANG J, BIEDERMANN KA, WOLF CR AND BROWN JM. (1993). Metabolism of the bioreductive cytotoxin SR 4233 by tumour cells: enzymatic studies. *Br. J. Cancer*, **67**, 321–325.

WORKMAN P. (1992). Bioreductive mechanisms. *Int. J. Radiat. Oncol. Biol. Phys.*, **22**, 631–637.

WORKMAN P. (1994). Enzyme-directed bioreductive drug development revisited. *Oncology Res.*, **6**, 461–475.

WORKMAN P AND STRATFORD IJ. (1993). The experimental development of bioreductive drugs and their role in cancer therapy. *Cancer Metas. Rev.*, **12**, 73–82.

WORKMAN P AND WALTON MI. (1986). Enzyme-directed bioreductive drug development. In *Selective Activation of Drugs by Redox Processes*, Adams GE, Beccia A, Fielden EM and Wardman P. (eds.) pp. 89–112. Plenum Press: New York.

ZEMAN EM, BROWN JM, LEMMON MJ, HIRST VK AND LEE WW. (1986). SR 4233: A new bioreductive agent with high selective toxicity for hypoxic mammalian cells. *Int. J. Radiat. Oncol. Biol. Phys.*, **12**, 1239–1242.